# Analysis of Ultra-Deep Pyrosequencing and Cloning Based Sequencing of the Basic Core Promoter/Precore/Core Region of Hepatitis B Virus Using Newly Developed Bioinformatics Tools

**DOI:** 10.1371/journal.pone.0095377

**Published:** 2014-04-16

**Authors:** Mukhlid Yousif, Trevor G. Bell, Hatim Mudawi, Dieter Glebe, Anna Kramvis

**Affiliations:** 1 Hepatitis Virus Diversity Research Programme, Department of Internal Medicine, University of the Witwatersrand, Johannesburg, Gauteng, South Africa; 2 Department of Medicine, Faculty of Medicine, University of Khartoum, Khartoum, Khartoum State, Sudan; 3 Institute of Medical Virology, National Reference Centre of Hepatitis B and D, Justus, Liebig-University of Giessen, Giessen, Hesse, Germany; Saint Louis University, United States of America

## Abstract

**Aims:**

The aims of this study were to develop bioinformatics tools to explore ultra-deep pyrosequencing (UDPS) data, to test these tools, and to use them to determine the optimum error threshold, and to compare results from UDPS and cloning based sequencing (CBS).

**Methods:**

Four serum samples, infected with either genotype D or E, from HBeAg-positive and HBeAg-negative patients were randomly selected. UDPS and CBS were used to sequence the basic core promoter/precore region of HBV. Two online bioinformatics tools, the “Deep Threshold Tool” and the “Rosetta Tool” (http://hvdr.bioinf.wits.ac.za/tools/), were built to test and analyze the generated data.

**Results:**

A total of 10952 reads were generated by UDPS on the 454 GS Junior platform. In the four samples, substitutions, detected at 0.5% threshold or above, were identified at 39 unique positions, 25 of which were non-synonymous mutations. Sample #2 (HBeAg-negative, genotype D) had substitutions in 26 positions, followed by sample #1 (HBeAg-negative, genotype E) in 12 positions, sample #3 (HBeAg-positive, genotype D) in 7 positions and sample #4 (HBeAg-positive, genotype E) in only four positions. The ratio of nucleotide substitutions between isolates from HBeAg-negative and HBeAg-positive patients was 3.5∶1. Compared to genotype E isolates, genotype D isolates showed greater variation in the X, basic core promoter/precore and core regions. Only 18 of the 39 positions identified by UDPS were detected by CBS, which detected 14 of the 25 non-synonymous mutations detected by UDPS.

**Conclusion:**

UDPS data should be approached with caution. Appropriate curation of read data is required prior to analysis, in order to clean the data and eliminate artefacts. CBS detected fewer than 50% of the substitutions detected by UDPS. Furthermore it is important that the appropriate consensus (reference) sequence is used in order to identify variants correctly.

## Introduction

The continued improvement of DNA sequencing technologies has led to the development of next generation sequencing (NGS) methods, including ultra-deep pyrosequencing (UDPS), which are capable of sequencing many thousands of nucleotides, quickly and at a low cost per nucleotide. These technologies have overcome the disadvantages of the traditional dye-terminating DNA sequencing technology developed by Frederick Sanger [1]. These disadvantages include the relatively high cost per nucleotide, in terms of money and time, and the fact that Sanger sequencing is only capable of detecting sequence variants, which are present in 20% or more of a quasispecies population [2], [3]. Moreover, NGS methods also overcome several of the drawbacks of cloning based sequencing (CBS), such as the time, money and expertise required to prepare samples, especially when a large number of clones is required [4]. NGS methods are used primarily for *de novo* or “shot-gun” sequencing of new or known genomes. This produces a very large number of short reads, which are then assembled to produce a complete sequence. Several algorithms and tools exist to process these short reads [5]. In addition to producing short reads, the pyrosequencing platform can be used for amplicon re-sequencing (UDPS). These longer reads are typically an amplicon covering a genomic region of interest. At present, the GS Titanium UDPS chemistry produces reads of approximately 400 bases in length.

Few bioinformatic tools, which are affordable and accessible to resource-constrained environments, are currently available to assist with the processing and analysis of amplicon re-sequencing data. The Roche AVA software (http://www.454.com/products/analysis-software/#amplicon-tabbing), although free of charge, can only be installed on a computer running a particular GNU/Linux distribution, and a number of commercial software packages cost several thousand US dollars for a single license. Alignment and visualization tools, which are used routinely for smaller datasets, are not suitable for datasets containing hundreds or thousands of reads. Additionally, many of these software solutions require a level of technical expertise, which many biological researchers may not possess.

Pyrosequencing is an error-prone technique [6]. Distinguishing between a true biological variant and an error (artefact) is a vital step in analysing pyrosequencing data. Although a number of studies discuss error correction in pyrosequencing data [6], [7], there is currently no consensus regarding the error threshold, which should be applied. Knowledge of well-characterized regions of a genome is important in order to develop tools to examine pyrosequencing data and to distinguish between artefacts and true variations.

Hepatitis B virus (HBV) displays remarkable sequence heterogeneity, with 9 genotypes (named A to I) currently recognized [8], [9]. The precore/core (PC/C) open reading frame (ORF) of HBV encodes for both the hepatitis B e antigen (HBeAg) and the core protein (HBcAg). This region is preceded by the basic core promoter (BCP) region, which controls transcription of both the PC/C mRNA and the pregenomic RNA (pgRNA) during the replication cycle [10]. The BCP/PC ORF overlaps the X ORF. HBeAg is a soluble, non-particulate protein that is secreted in the serum or expressed on the surface of the hepatocyte [11], [12]. Conventionally, HBeAg expression is an indicator of active HBV infection and on-going viral replication [12]. However, HBeAg expression may be reduced or completely suppressed by various viral mutations, even in the presence of viral replication. Mutations in two regions may affect HBeAg expression: precore mutations (for example, G1896A) [13] and BCP mutations (for example, A1762T/G1764A) [14]. The viral capsid is composed of HBcAg [15]. Mutations may occur more frequently in N-terminal or central region of the core protein, which does not overlap other reading frames [16].

Using a segment of this well-characterized BCP/PC/C region of HBV as a model, the objectives of this study were to:

- develop bioinformatics tools to explore UDPS data,
- test and use them to determine the optimum error threshold, and
- compare results between UDPS and CBS using HBeAg-positive and –negative sera infected, with either genotype D or E.

## Materials and Methods

### Sample Selection

Written informed consent was obtained from all participants and the consent was approved by the Sudanese Ministry of Health, who gave permission for the sera to be used for research purposes. The Human Ethics Committees of the University of the Witwatersrand and the University of Khartoum approved the study. Four serum samples were selected from our previous study on HBV from monoinfected individuals, where the HBV genotype was determined using phylogenetic analysis [17]. Sample #1 was HBeAg-negative and infected with genotype E of HBV (GenBank, KF170783), sample #2 was HBeAg-negative, genotype D (KF170739), sample #3 was HBeAg-positive, genotype D (KF170740) and sample #4 was HBeAg-positive, genotype E (KF170788).

### Wet Laboratory Work

#### Ultra-Deep Pyrosequencing (UDPS)

A region of the HBV genome (1653–1959 from *Eco*R1 restriction site) was amplified using a slight modification of a previously described method [18]. Primers 1606 (+) and 1974 (−) were used for the first round PCR, and 1653 (+) and 1959 (−) for the second round PCR. The first round PCR was followed by gel-purification using Zymoclean Gel DNA Recovery Kit (Zymo Research Corp, Irvine, CA, USA). For the second round PCR, modified primers, which were ligated to adaptors and tags, were used (Table 1). Following second round PCR, the amplicons were gel-purified and subjected to UDPS in the forward direction on the Roche 454 GS Junior platform (454 Life Sciences, Roche Company, Switzerland), which provided reads covering the region of interest (coordinates 1653–1959). The UDPS sequencing data has been submitted to the GenBank SRA database, as BioProject accession: PRJNA239442 and the following are the BioSample accessions: SAMN02664575, SAMN02664576, SAMN02664577, SAMN02664578.

**Table 1:** Sequences of HBV-specific primers, tags and adaptors used for ultra-deep pyrosequencing.

	Position from *Eco*RI site	HBV specific target sequence	Tag sequences¶	Adaptor sequences€
**PCR 1**	1606 (+) [1606–1625]	5′-GCATGGAGACCACCGTGAAC-3′	No tag	No adaptor
	1974 (−) [1974–1955]	5′GGAAAGAAGTCAGAAGGCAA-3′	No tag	No adaptor
**PCR 2**	1653 (+) [1653–1672]	5′-CGTATCGCCTCCCTCGCGCCATCAG-3′	ACACGACGACT^1^	CAT AAG AGG ACT CTT GGA CT
			ACACGTAGTAT^2^	
			ACACTACTCGT^3^	
			ACGACACGTAT^4^	
	1959 (−) [1959–1940]	5′-CTATGCGCCTTGCCAGCCCGCTCAG-3′	No tag	GGC AAA AAC GAG AGT AAC TC

¶ Short specific sequences used to label the different samples: ^1:^ sample #1; ^2:^ sample #2; ^3:^ sample #3; ^4:^ sample #4.

€ Short specific sequence used to ligated onto the ends of the fragments. These adaptors provide priming sequences for both amplification and sequencing of the sample-library fragments.

#### Cloning Based Sequencing (CBS)

After nested PCR, the 307 nucleotide amplicon (1653–1959 from *EcoR*1 site) was gel-purified and cloned into pTZ57R/T vector (55 ng/µl) using Instaclone PCR Cloning Kit (Fermentas, Waltham, MA, USA), and transformed into TOP10 *Escherichia coli* (Invitrogen, Carlsbad, CA, USA). The transformants were grown on Ampicillin plates. Positive clones were identified by restriction fragment length polymorphism (RFLP) assay. At least 20 clones per sample were sequenced by direct sequencing, using a BigDye Terminator v3.0 Cycle Sequencing Ready Reaction Kit (Applied Biosystems., Foster City, USA) on an ABI 3130XL Genetic Analyzer (Applied Biosystems). The sequencing primer used was M13 forward (5′-GTAAAACGACGGCCAGT-3′). A phylogenetic tree was generated as described previously [17]. Clone sequences have been deposited in GenBank: Genotype D: KJ496256-KJ496297, Genotype E: KJ496206-KJ496255.

### Dry Laboratory Work

#### Data pre-processing

**Figure 1:**
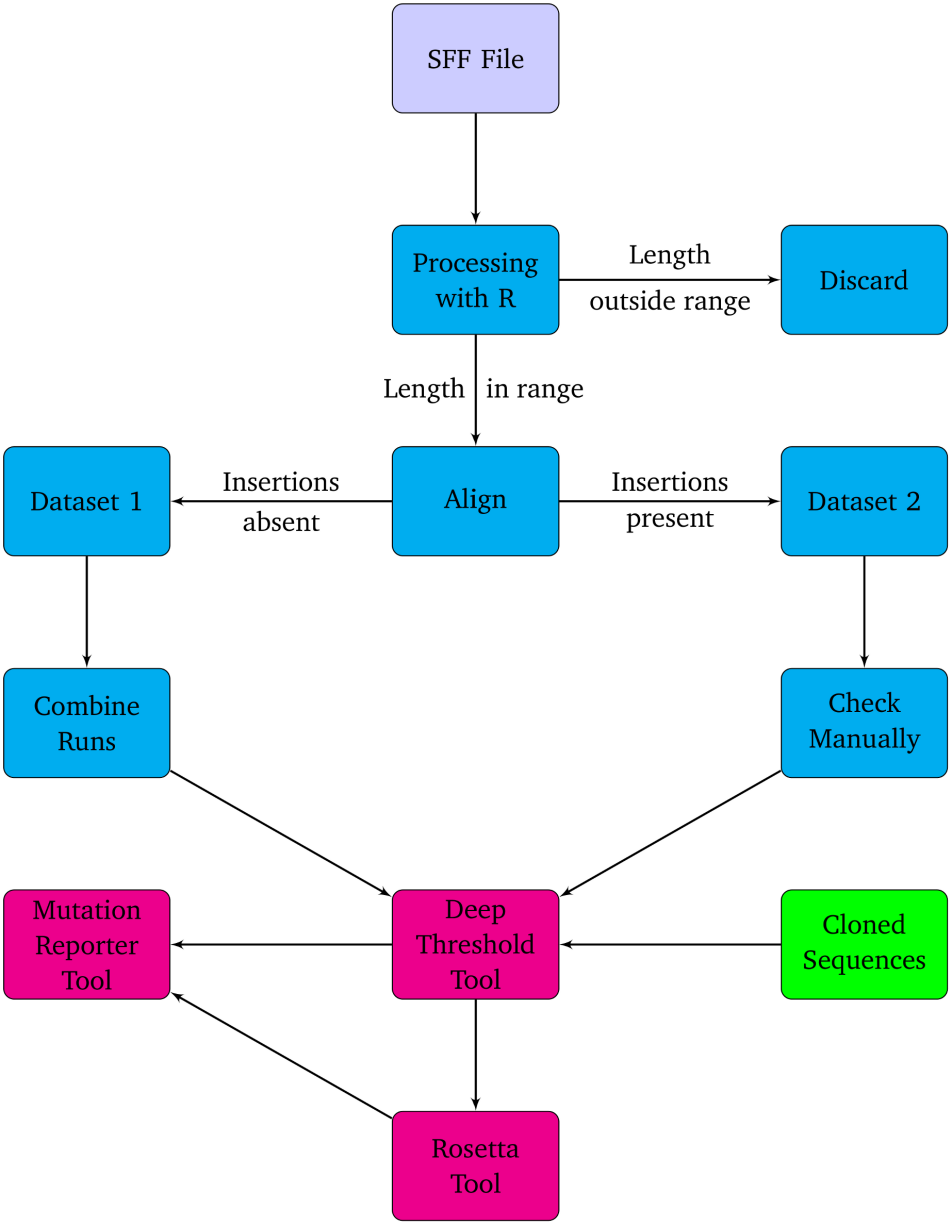
Flow diagram of the analysis procedure used for the UDPS data.

UDPS data for three sequencing runs, for each of the four samples, was processed and analyzed as shown in the flow diagram (Figure 1). The data from each run, for each sample, was processed individually. Separate binary standard flowgram format (SFF) files were opened in the R statistical programming language [19], using the “raw” clip-mode parameter (which does not perform any clipping or trimming) of the “rSFFreader” library [20]. Sequence data were searched for the forward and reverse primer sequences and the adaptor sequence for verification. Sequence lengths in each file were plotted and examined statistically (*data not shown*).

The distribution of all sequence lengths was examined and a length range was selected, which excluded reads with very low counts. Several Linux command-line BASH scripts and Python programming language scripts (*available on request*) were written to include only reads within a specified length range (between 330 to 360 nucleotides) for further processing. A genotype D reference sequence (GU456684) was then added to each dataset, and the file was aligned with the Muscle program [21]. Each alignment was then processed by a Python script, which scanned the reference sequence in the alignment and removed any reads from the alignment with an insertion (a residue aligned with a gap in the reference sequence). In the remaining alignment (excluding reads with insertions), positions (columns), containing only gaps, were collapsed and this alignment was “Dataset 1”. The repeated runs for all “Dataset 1” sequences for each sample were then combined into one dataset, the final “Dataset 1”. The file containing reads with insertions was “Dataset 2” for each run and these were processed individually because of variable read lengths, as a result of insertions at different positions in the reads.

#### Development of deep threshold tool

**Figure 2:**
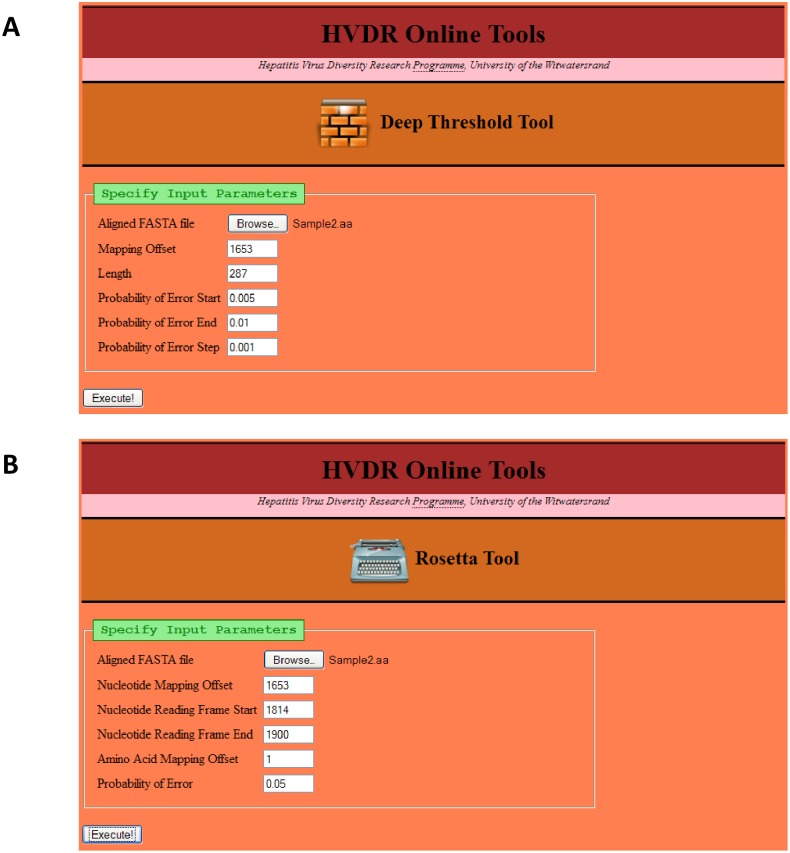
The input pages of the bioinformatics tools (A) “Deep Threshold Tool”. The first field specifies the input FASTA file. Fields are available for the user to specify the nucleotide offset mapping of the first position in the input file, the number of nucleotides (length) to process, the starting and ending probabilities of error to examine, and the probability of error increment (step) to use. (**B**) “Rosetta Tool”. The first field specifies the input FASTA file. Fields are available for the user to specify the nucleotide offset mapping of the first position in the input file, the position of the first in-frame nucleotide of the coding region of interest, the last in-frame nucleotide of the coding region of interest, the amino acid offset of the first amino acid in the coding region of interest, and the probability of error to use.

For pyrosequencing data of human immunodeficiency virus (HIV), a probability of error, ranging from 0.5% to 1%, has been used [6]. In the present study, using HBV data, a web-based tool (the “Deep Threshold Tool”) (http://hvdr.bioinf.wits.ac.za/tools/) was developed to examine the number of errors in each position (column) in an alignment, depending on the probability of error value. In order to examine the number of errors, the tool requires an input alignment in FASTA format, the lower and upper bounds of the probability of error, and an increment value (Figure 2A). A nucleotide mapping offset can be specified, so that the resulting output coordinates reflect the correct position of the sequence in the entire genome. Potentially untidy ends of reads (such as the reverse primer region) can be excluded from the analysis by specifying a length shorter than the sequence length.

#### Statistical calculation of the threshold

A nucleotide was considered an “error” if its frequency in a column in the alignment was less than the threshold, which was determined as follows. An expected frequency of ***E = probability of error×read depth (R)*** was used. A Pearson’s χ^2^ test statistic was calculated as follows:
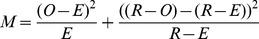
with ***O*** being the observed value, starting at 1. If ***M*** was less than the χ^2^ distribution (with α = 0.05 and one degree of freedom), then ***O*** was incremented by a value of one and the test was repeated. The value for *O* at which the χ^2^ distribution was exceeded, was considered the threshold value (count). This threshold was calculated for each position in the alignment. Any nucleotide with a frequency below this threshold was considered an error or artefact.

#### Development of rosetta tool

Amino acid data were examined using the newly-developed “Rosetta Tool”. This tool requires the same input file as the “Deep Threshold Tool “. It also requires a nucleotide offset mapping and the start and end positions of a protein region. This does not have to include the position of the start or stop codon; any region of a protein can be processed, as long as the number of nucleotides specified by the range is a multiple of three. The probability of error at which the data must be analyzed is also required (Figure 2B).

## Results

A total of 10952 reads were generated on the 454 GS Junior platform for the three runs for all four samples. Of these, 9738 reads (88.9%) were included in the study (2002, 3049, 1955 and 2732 reads for samples 1, 2, 3 and 4, respectively) and 1214 reads (11.1%), which were considered either too short or too long, were excluded. These 9738 reads were split into Dataset 1 (8967 reads, 92.1%) and Dataset 2 (771 reads, 7.9%) (Figure 1). Ninety-two clones were generated for all four samples: 23 clones for sample #1, 22 for sample #2, 20 for sample #3 and 27 for sample #4.

### Deep Threshold Tool Output

**Figure 3:**
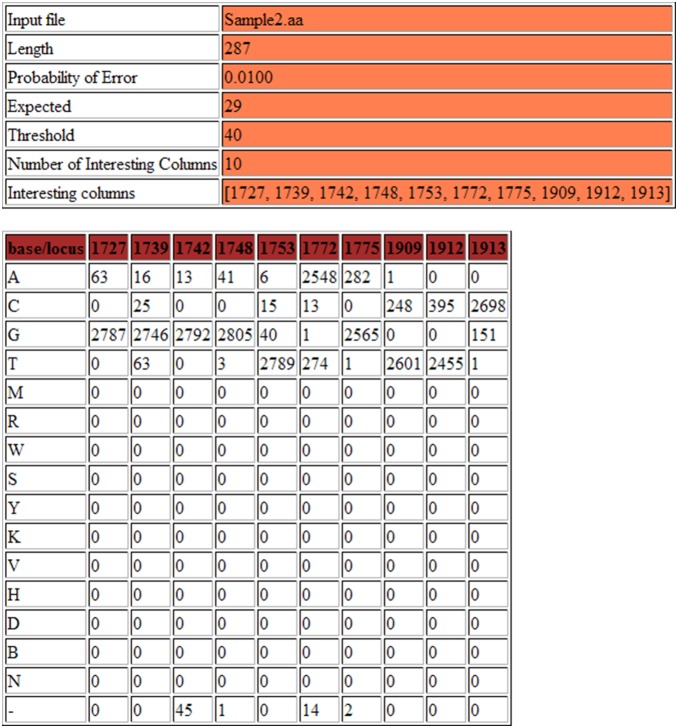
An example section of the output from the “Deep Threshold Tool”, showing the two tables of output provided for each probability of error examined. The “expected” and “threshold” counts are shown in the top table, as well as the number of interesting columns (those columns containing at least one mutation at above-threshold frequency), and a list of the interesting columns. The bottom table provides detailed output, showing the number of each residue occurring in each interesting column.

**Figure 4:**
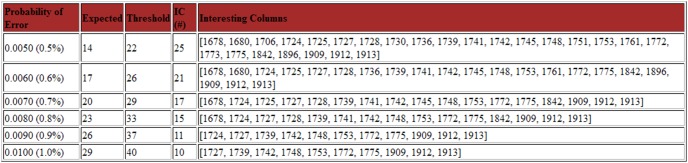
The first of two summary output tables provided by the “Deep Threshold Tool”. For each probability of error in the range specified, the expected and threshold values are shown, the number of interesting columns (IC) and the list of these interesting columns. This table provides a summary of the output provided in the previous tables (Figure 3 *top*).

**Figure 5:**
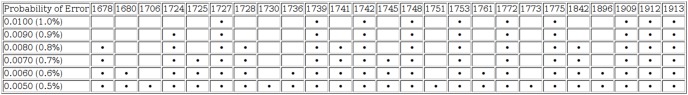
The second of two summary output tables provided by the “Deep Threshold Tool”. For each probability of error in the range specified (shown in reverse order in this table), a bullet is shown in the corresponding column of the table for each interesting column at which at least one mutation occurred at above-threshold frequency. This table can be consulted to determine the probability of error, which should be used on a given dataset. In this example, the well-characterized positions 1753, 1773 and 1896 are examined, and a probability of error of 0.005 selected, as this is the highest probability of error at which above-threshold mutations at the three positions are detected.

The output page generated by the Deep threshold Tool includes a table for each increment of the probability of error (Figure 3), which shows the distribution of nucleotides at all columns at which at least one base can be considered an “error”. Because a nucleotide was considered an “error”, if its frequency in a column in the alignment was less than the threshold, any variation above the threshold was considered a legitimate variant for that probability of error (Figure 4). Figure 5 summarizes the results graphically. This summary table was consulted and the lowest probability of error, at which established, well-characterized variants are still detected, was selected. In the present study, the lowest probability of error at which substitutions at positions 1753, 1773 and 1896 are still evident was 0.5%, and this was taken to be our probability of error value.

### Rosetta Tool Output

**Figure 6:**
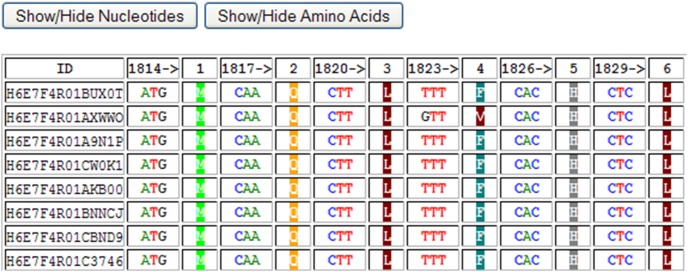
The first output table of the “Rosetta Tool”, showing codon (triplets), followed by single-letter translated amino acids, for each read in the input FASTA alignment. This alignment can be used to easily locate mutations of interest and to locate synonymous and non-synonymous mutations. The visibility of the nucleotide and/or amino acids columns can be toggled on or off, as required.

**Figure 7:**
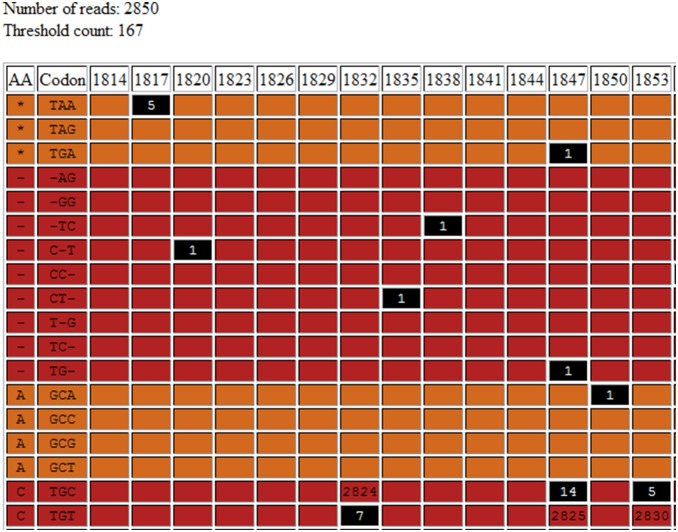
The second output table of the “Rosetta Tool” (truncated), showing the frequency of each possible codon (triplet) and where it occurs in the alignment. Frequencies shown with a black background occurred at below-threshold levels and therefore can be disregarded.

**Figure 8:**
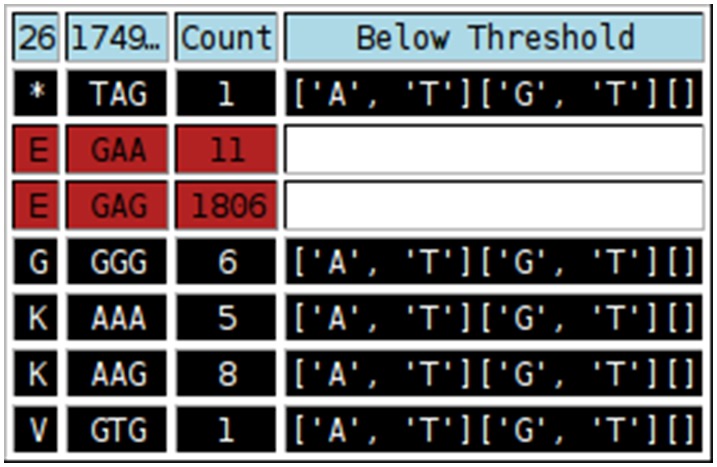
Examples of the final series of tables output by the “Rosetta Tool”, showing details of the codons (triplets) and amino acids occurring at each position in the alignment. Cells with black backgrounds indicate where at least one nucleotide in the triplet occurred at below-threshold levels. These rows can be disregarded. The “Below Threshold” column lists the residues, for each position of the codon (indicated by the square brackets), which were below the. threshold.

Alignments generated from direct sequencing, UDPS or CBS can also be submitted to the Rosetta Tool. This would typically be done in order to make use of the nucleotide/amino acid alignment viewer component of the tool. The tool produces a number of output tables (Figures 6–8). Figure 6 is an alignment showing each codon followed by the amino acid. Amino acids have been colour-coded according six different categories: Aliphatic (Glycine, Alanine, Valine, Leucine and Isoleucine), Hydroxyl (Serine, Cysteine, Threonine and Methionine), Cyclic (Proline), Aromatic (Phenylalanine, Tyrosine and Tryptophan), Basic (Histidine, Lysine and Arginin) and Acidic (Aspartate, Glutamate, Asparagine and Glutamine). The display of nucleotides or amino acids can be toggled on or off for ease of reference. Figure 7 shows the distribution of each residue at each position at which at least one residue is considered an error. Such error residue counts are highlighted with a black background for reference. Figure 8 contains separate tables for each codon at which at least one residue is an “error”, and shows the distribution of codons and amino acids at this position. Synonymous and non-synonymous mutations can be differentiated. Rows containing substitutions occurring below the threshold, “error” nucleotides are highlighted with a black background.

In order to analyze the data downstream, the Rosetta Tool produces a “masked” data file, which is generated by replacing all “error” residues in the nucleotide alignment, with an “X” character. This alignment is then be translated into amino acids, with an amino acid of “X” used whenever at least one “X” character per codon occurs. Both the nucleotide and amino acid masked files can be downloaded in FASTA format.

Using the selected probability of error of 0.5%, masked files were generated and the UDPS data were then analyzed using the two newly developed tools and the Mutation Reporter Tool [22].

### Analysis of Pyrosequencing Reads

**Figure 9:**
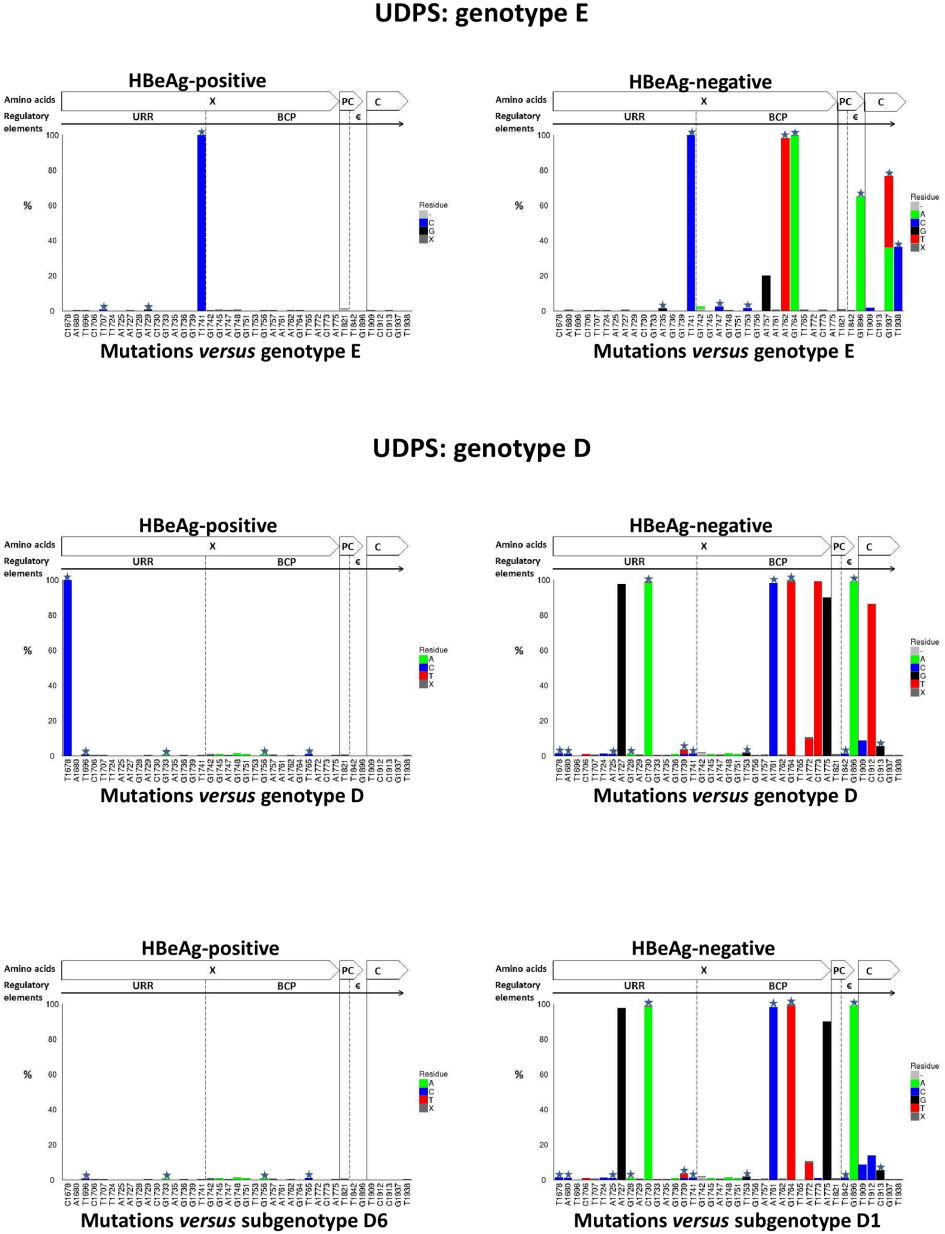
Graphs showing mutation distribution of the UDPS data at the nucleotide level using either genotype E or D consensus sequence as the reference. A star indicates a non-synonymous mutation. The graphs were built using the Mutation Reporter Tool [22].

Each sample in Dataset 1 was then analysed using the newly developed “Deep Threshold Tool” and a probability of error of 0.5% was selected, because this was the lowest probability of error at which all three well characterized mutations (T1753G/C, T1773C and G1896A) were present. The resulting threshold (count) value will differ depending on the number of reads (depth) in each file, for a given probability of error. For each sample, output of the “Deep Threshold Tool” lists the loci detected at above threshold value and these were then analyzed using the Mutation Reporter Tool, with a reference motif being the corresponding consensus sequences for each genotype or subgenotype. The distribution of substitutions at the nucleotide level in the BCP/PC/C region varied between samples, depending on the HBV genotype and HBeAg status (Figure 9). At 0.5% probability of error or above, substitutions were identified at 39 unique positions in the four samples:31 in the X region (1674 to 1838 from the *Eco*R1 site; 165 nucleotides), three in the PC region (1814 to 1900; 87 nucleotides) and five in the core region (1901 to 1939; 39 nucleotides) (Figure 9). Ten of the 39 positions were present in at least two samples.

Based on the fact that direct sequencing is capable of detecting substitutions occuring in ≥20%, of the quasispecies population substitutions were classified as high frequency (≥20%) and low frequency substitutions (<20%). High frequency substitutions were found at 11 positions and low frequency at 28 positions.

A consensus of genotype E was used to identify substitutions in genotype E (samples #1 and #4). The T1741C substitution was detected in both samples at a high frequency, regardless of the HBeAg status, while the following substitutions: A1757G, A1762T, G1764A, G1896A, G1937A/T and A1938C, were found at a high frequency in HBV from a HBeAg-negative patient (sample #1) (Figure 9). Substitutions A1735G, G1742A, A1747C, T1753C and T1909C were found at a low frequency in sample #1, and T1707C was found at a low frequency in sample #4 (Figure 9).

Similarly, when the genotype D sequences (samples #2 and #3) were compared to their corresponding consensus sequence, 1678T was found in sample #2 and 1678C in sample #3. The consensus of genotype D had 1678T. From phylogenetic analysis carried out in our previous study, HBV from sample #2 belongs to subgenotype D1 and from sample #3 to subgenotype D6 [17]. The consensus of subgenotype D1 has T at 1678 and that of subgenotype D6 has C. Therefore, when sample #3 was compared to the consensus of subgenotype D6, only low frequency substitutions were detected (T1696C, G1733A, G1745A, G1748, G1751A, G1756A and T1765C) (Figure 9). When the reference sequence was changed from the D to D1, the mutation pattern of sample #2 (subgenotype D1), changed (Figures 9). Using either reference sequence D or D1, the following substitutions were detected with high frequency: A1727G, C1730A, A1761C, G1764A, A1775G and G1896A, whereas the frequency of 1773T and 1912T decreased when using D1 instead of D as the reference sequence (Figure 9). The following substitutions relative to D1, occurred in sample #2 at low frequency: T1678C, A1680C, C1706T, T1724C, A1725C, G1728A, G1736A, G1739C/T, T1741C, G1745A, G1748A, G1751, T1753G, A1772T, T1773C, T1842C, T1909C, T1912C and C1913G.

Summarizing the above, in the four samples substitutions were identified at 39 unique positions. Sample #2 (HBeAg-negative, genotype D) had substitutions in 26 positions, followed by sample #1 (HBeAg-negative, genotype E) in 12 positions, sample #3 (HBeAg-positive, genotype D) in 7 positions and sample #4 (HBeAg-positive, genotype E) in only four positions. The ratio of nucleotide substitutions between isolates from HBeAg-negative and HBeAg-positive patients was 3.5∶1. Moreover, genotype D isolates showed greater variation in the X, PC and core regions, compared to genotype E isolates, with the two genotype D samples having 33 substitutions compared to the 16 detected in the genotype E samples.

The “Rosetta Tool”, which was developed as part of this study, was used to analyze sequence data at the amino acid level. Substitutions identified at the nucleotide level were translated into amino acids and classified as synonymous or non-synonymous. Fourteen substitutions, 12 in the X region and 2 in the C region, were synonymous. Twenty-five, 19 in the X region, three each in the PC and C regions, were non-synonymous mutations. All non-synonymous mutations occurred within single, non-overlapping reading frames (1653 to 1814, and 1839 to 1939 from the *Eco*R1 restriction site), and the region between the start of the PC and the end of the X (1814 to 1838) was completely conserved in all ultradeep pyrosequences.

Most of the 21 insertions found in Dataset 2 occurred within homopolymeric regions and were therefore considered to be PCR or pyrosequencing artefacts [23].

### Analysis of CBS and Comparison to UDPS

**Figure 10:**
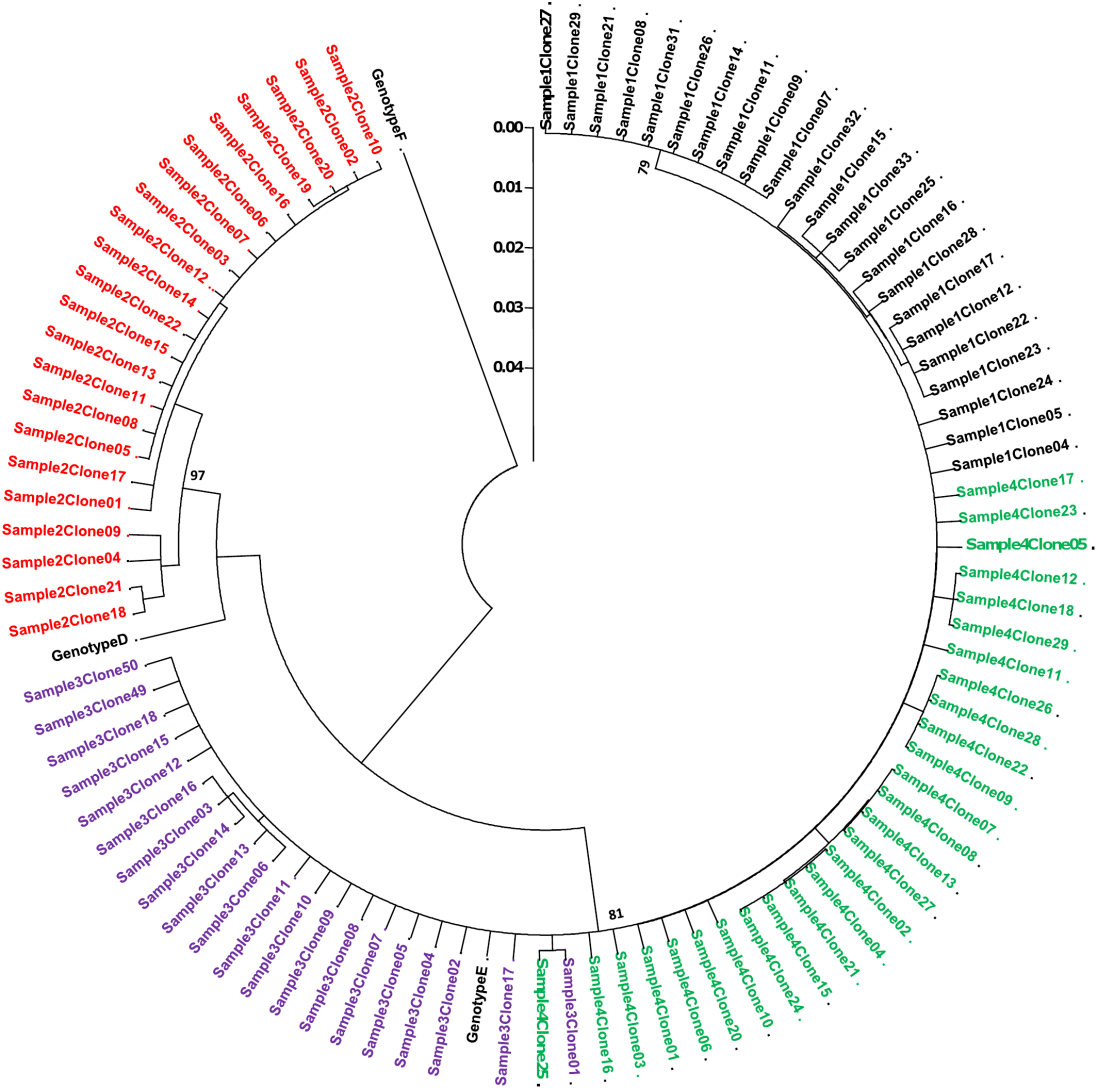
A rooted phylogenetic tree of 92 cloned BCP/PC sequences (position 1653 to 1939 from *EcoR*I site) from four serum samples. Sample #1 was HBeAg-negative and infected with genotype E of HBV, sample #2 was HBeAg-negative, genotype D, sample #3 was HBeAg-positive, genotype D and sample #4 was HBeAg-positive, genotype E. Bootstrap statistical analysis was performed using 1000 datasets, indicated as percentages on the nodes. The letters, D and E, represent the genotypes.

At least 20 clones were generated per sample. The BCP/PC region sequenced is relatively short and does not differentiate genotypes D and E following phylogetic analysis. Both identical and multiple clones were generated, with HBV from HBeAg-negative sera showing greater divergence (Figure 10).

**Figure 11:**
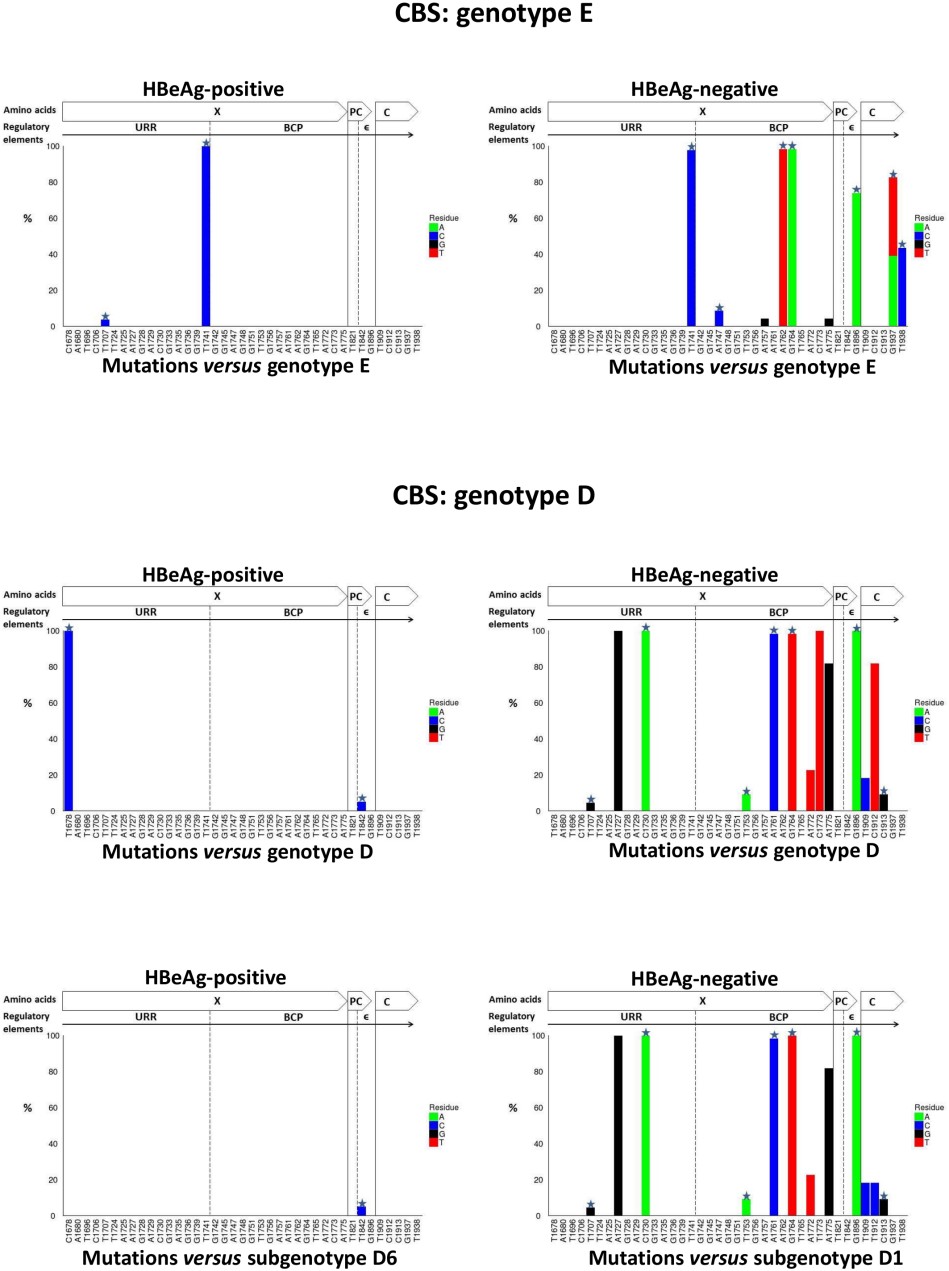
Graphs showing mutation distribution of the CBS data at the nucleotide level using either genotype E or D consensus sequence as the reference. A star indicates a non-synonymous mutation. The graphs were built using the Mutation Reporter Tool [22].

CBS data was analyzed at the 39 loci, previously recognized by UDPS, using the Mutation Reporter Tool and a consensus sequence for each genotype/subgenotype as the reference sequence. In the four samples, substitutions at 18 of the 39 positions (46.2%) were detected by CBS (Table 2) (Figure 11). CBS detected all high frequency substitutions but only 25% (7/28) of the low frequency substitutions (Table 2). Moreover, the following nucleotide substitutions were detected in different samples by either UDPS or CBS, at position:

**Table 2:** Mutations detected by CBS and UDPS.

Frequency	Detected by	Mutations
High (≥20%)	UDPS and CBS	A1727G,C1730A,T1741C,A1757G,A1761C,A1762T,G1764A/T,A1775G,G1896A,G1937A/T,T1938C
Low (<20%)	UDPS and CBS	T1707C, A1747C, T1753G, A1772T, T1909C,T1912C, C1913G
Low (<20%)	UDPS	T1678C, A1680C, T1696C, C1706T, T1724C, A1725C, G1728A, A1729G, G1733A, A1735G, G1736A, G1739C/T, T1741C, G1742A, G1745A, G1748A, G1751A, T1753C, G1756A,T1765C, T1733C, T1842C

1. 1707 by CBS in sample #2 and by UDPS in sample #4,
2. 1775 by CBS in sample #1 and by UDPS in sample #2 and
3. 1842 by CBS in sample #3 and by UDPS in sample #2.

For the four samples, CBS detected only 14 of the 25 non-synonymous mutations, detected by UDPS (58.3%).

## Discussion

The aims of this study were to build bioinformatics tools to assist in determining the threshold at which pyrosequencing data should be analyzed, and to compare quasispecies distributions obtained using UDPS and CBS, and compare results between UDPS and CBS using HBeAg-positive and –negative sera infected, with either genotype D or E.

Direct (Sanger) sequencing produces a single “read” for each sample. After curating the sequence and resolving ambiguous bases, the sequence is ready for further downstream processing. Whilst UDPS, which generates several thousand reads per sample, is a powerful technology, the analysis of the read data before downstream processing is critical. The depth of coverage provided by UDPS is also one of its shortcomings, as the data needs to be carefully curated for errors (artefacts), which may have been introduced by the PCR amplification and/or the sequencing process [2], [23]. The increased sensitivity of the platform to detect thousands of reads also means that it may generate such artefacts.

A probability of error of between 0.5% and 1% for UDPS has been used previously for HIV samples [6]. Subsequent studies on HBV sequence data have either used the same probability of error, or have not reported details of this component of the analysis [2], [3], [24]. The probability of error, which is used, will influence the downstream detection of variants. As such, selecting an appropriate probability of error is an essential step in the analysis. In response to the lack of consensus in selecting a probability of error and determining a threshold, we developed an online bioinformatics tool to explore this aspect of the analysis. The “Deep Threshold Tool” provides the researcher with detailed output of variation at different probabilities of error. The analysis is objective and repeatable, and the selected probability of error can be reported and defended. Data for a project can be processed by the tool, so that a probability of error can be selected for that specific project, organism or assay. Using a fixed, predetermined probability of error for the UDPS platform as a whole is overly-broad and too general, as it is not possible to indicate how a particular probability of error would be applicable to a different organism, genomic region or investigation. Using the “Deep Threshold Tool” developed in the present study, a probability of error of 0.5% was selected for the BCP/PC/C region of HBV, which agrees with previous reports for HIV [6].

The output must be interpreted in light of existing biological knowledge of the variation known to occur in the sequenced region. The tool is objective and outputs results for different probabilities of error “blindly”. There is no “right answer” or absolute correct threshold, as we cannot possess complete knowledge of all the stochastic processes, from the sample to the PCR to the sequencing platform to the sequence results. Variation may be introduced at the various PCR stages, rather than by the sequencing hardware itself [23]. What we can do, however, is to interrogate these data at different probabilities of error, and make an informed decision on which value to select. It is important that the method used to process and curate the UDPS data, as well as any numerical values used (such as probability of error or threshold), be reported in all UDPS studies. Failure to provide this level of detail makes it difficult to accurately assess and relate any results reported.

The emergence of G1896A mutation in the PC region is known to be associated with HBeAg seroconverion [13]. The presence of wild-type (G) at 1896 in sample #1 and sample #2,which were isolated from HBeAg-negative patients, confirms the ability of UDPS to detect minor populations, which may not be detected by Sanger sequencing [25], [26]. Similar results have been reported in more recent HBV studies. The HBV population from HBeAg-positive sera showed a high percentage of stop codon mutations in the precore region, while isolates from HBeAg-negative carriers had a low percentage of wild-type residues at codon 28 [24].

Although the selection of genotype D samples was random, we later discovered that sample #3 belonged to subgenotype D6, while sample #2 belonged to subgenotype D1. As illustrated in Figures 9 and 10, knowledge of the genotype and subgenotype of HBV is important when determining the presence of mutations. Depending on the reference or consensus sequence used, the variant at a particular position, may either represent the signature of a particular subgenotype or be a legitimate mutation. Therefore, where possible, a consensus sequence of the genotype or subgenotype should be used, to ensure that variants are examined in the appropriate context.

Six mutations (A1757G, A1762T, G1764A G1896A, G1937A/T and A1938C) were found in high frequency (>20%) in sample #1, genotype E isolated from a HBeAg-negative patient. The G1896A mutation is known to create the stop codon at amino acid 28 and to abrogate HBeAg expression [13], while the double mutation A1762T/G1764A is known to down-regulate the transcription of precore mRNA that is translated into HBeAg [14]. Although A1757G is a synonymous mutation and thus has no effect on the protein sequence, it overlaps *cis*-regulatory elements within the basic core promoter. In the present study, 1757G was found to be associated with A1762T/G1764A. This association has also been shown by others, who found that chronic hepatitis patients infected with HBV with 1757G/1762A1764A had higher HBV DNA levels compared to patients infected with the wild-type 1757A/1762T1764A [27]. Moreover, A1757G was found to in HCC patients infected by genotype C [28]. Non-synonymous mutations G1937A/T and T1938C within the core region occurred at a high frequency (Figure 9). These mutations are located within a T-cell epitope, which is an important component of the host’s immune response to HBV infection [29]. These two mutations have recently been reported in strains of HBV genotype B isolated from Taiwanese patients [30]. Other substitutions (T1707C, A1735G, A1747C and T1909C) were found at low frequencies (<20%) and have not been reported in previous studies.

In sample #2 (genotype D isolated from HBeAg-negative), mutations A1727G, C1730A, A1761C, G1764A, A1775G and G1896A were detected at high frequency. A1727G and C1730A are located in the Enhancer II region and have been detected in cirrhotic patients [28] and are associated with reduced HBcAg expression and HBV DNA levels in the liver [31]. A1761C has previously been detected within a mutational motif (1761–1766) in isolates from patients with cirrhosis and chronic hepatitis [32]. The A1775G is associated with loss of HBeAg in Taiwanese children [33]. T1678C, G1753A and T1773C, which were found in the minority of the quasispecies population, have previously been associated with severity of HBV infection and progression to HCC [28], [34].

The following substitutions were found as minor populations and have not previously been documented. In HBV from HBeAg-negative samples: A1735G, G1742A, A1747C and T1909C in genotype E and A1680C, C1706T, T1724C, A1725C, G1728A, G1736A, G1739C/T, G1751A, A1772T, T1842, T1909C, T1912C and C1913G in genotype D (Figure 9) and in HBeAg-positive samples: T1696C, G1733A and G1751A in genotype D and T1707C in genotype E. Mutations G1745A and G1748A were found in both HBeAg-negative and HBeAg-positive genotype D samples. It is possible that these have not previously been detected because direct (Sanger) sequencing can only detect variation that occurs in 20% or more of the population. More extensive studies may reveal the relevance of these minor variants.

The genotype E isolates were found to harbour fewer mutations in the X, PC and core regions compared to genotype D, which is in agreement with previous studies showing low genetic diversity of genotype E [35], [36]. Furthermore, a greater number of mutations were found in HBeAg-negative samples of both genotype D and E compared to HBeAg-positive samples. It was reported that the frequency of HBV mutations is higher in HBeAg-negative patients, this is as a result the immune response of the host against the virus before the loss of HBeAg [37]. However, because only four samples, belonging to the two genotypes from HBeAg-positive and HBeAg-negative samples, were analyzed, additional samples would be required before any firm conclusions can be reached about the differences in nucleotide divergences between these genotypes from HBeAg-positive and –negative sera.

In this study, where 9738 sequence reads were generated by UDPS, 39 unique positions were detected by UDPS, while only 18 (46.2%) of these position were detected by CBS. High frequency substitutions were found in 11 positions and were all detected by CBS, whereas only 6/28 (25%) low frequency substitutions were detected by CBS (p<0.05) (Figures 9 and 10).

Although the testing of the tools was done on a small sample set and the findings cannot be generalized, it is evident that the data generated by the increased read-depth provided by UDPS should be approached with caution. Appropriate curation and examination of the reads are required to ensure that artefacts are not interpreted as variants. Moreover, identification of variants must be performed against a suitable reference or consensus sequence, as a “mutation” of interest may simply be a known signature or variant when examined in the correct genotypic or subgenotypic context. UDPS detected a greater number of substitutions than CBS. Relative to CBS, UDPS is cheaper to undertake, both in terms of time and expense. However, without rigorous and careful examination and interpretation of read data, the results generated by UDPS may be misleading. As illlustrated in the present study, a thorough knowledge of the genome of interest and its known variants is essential in order to accurately and reliably interpret the high resolution read data generated by UDPS.
